# Surgery for ulnar nerve decompression and drainage of a caseous granuloma in a neural form of leprosy in children

**DOI:** 10.1002/ccr3.7963

**Published:** 2023-10-05

**Authors:** Fernando Henrique Morais de Souza, Luiz Euripedes Almondes Santana Lemos, Bruno Rafael Sousa Rosado, Raiza Rafaela Borges De Oliveira, Mayle Gomes Ferreira de Araújo, Rhuann Pontes dos Santos Silva, Nivaldo Sena Almeida, Hildo Rocha Cirne Azevedo‐Filho

**Affiliations:** ^1^ Department of Neurosurgery Hospital da Restauração Recife Brazil; ^2^ Neurosurgery Resident Hospital da Restauração Recife Brazil; ^3^ Catholic University of Pernambuco Recife Brazil

**Keywords:** deformity, leprosy, neurosurgery, peripheral nerve decompression

## Abstract

**Key Clinical Message:**

The findings in the literature, as well as those described in this study, emphasize the need for systematic and longitudinal care for patients with neglected diseases during and after treatment, mainly in low‐middle income countries.

**Abstract:**

Leprosy is a chronic, granulomatous, mycobacterial infection caused by mycobacterium leprae, affecting the skin and peripheral nervous system. We present a case of a 13‐year‐old child with leprosy for more than a year, indicating decompression of the ulnar nerve. During surgery, an intraneural large caseous granule was evidenced.

## Graphical abstract

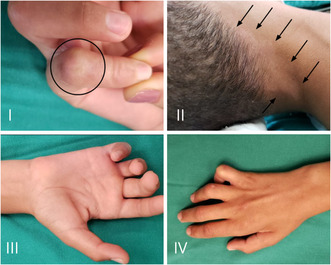

## 1 INTRODUCTION

Leprosy is a chronic, granulomatous, mycobacterial infection caused by *Mycobacterium leprae*, affecting the skin, peripheral nervous system, and occasionally other organs and systems, representing a serious public health problem.^1^ Three countries account for 83% of leprosy cases worldwide: India (58%), Brazil (16%), and Indonesia (9%).^2^ The main route of elimination of bacilli is the upper airway of patients with multibacillary forms.^3^

It is more common in adults, but when in children and adolescents, it indicates active circulation of bacilli representing a flawed and deficient health system.^4^ Due to the long incubation period of the disease, in highly endemic regions, many children are exposed early to a high bacillary load, which leads to a large number of children developing the disease in the future.

Among children, the most affected age group is between 10 and 14 years old, which can be explained by the long incubation time of the disease, with a similar incidence among boys and girls.^5^

The most common symptom is loss of sensation and pain followed by progressive motor deficit. Motor deficit causes claw hand and foot drop. Loss of sensation leads to ulcers and trophic lesions.^6^

The involvement of neurological disease with indication for surgery in children under 15 years of age is quite rare and there are few reports in the literature.

We present a case of a 13‐year‐old child with leprosy for more than a year, envolving with significant motor deficit and deformity in the right hand, indicating decompression of the ulnar nerve. During surgery, the presence of an intraneural large caseous granule was evidenced.

## 2 CASE REPORT

**FIGURE 1:**
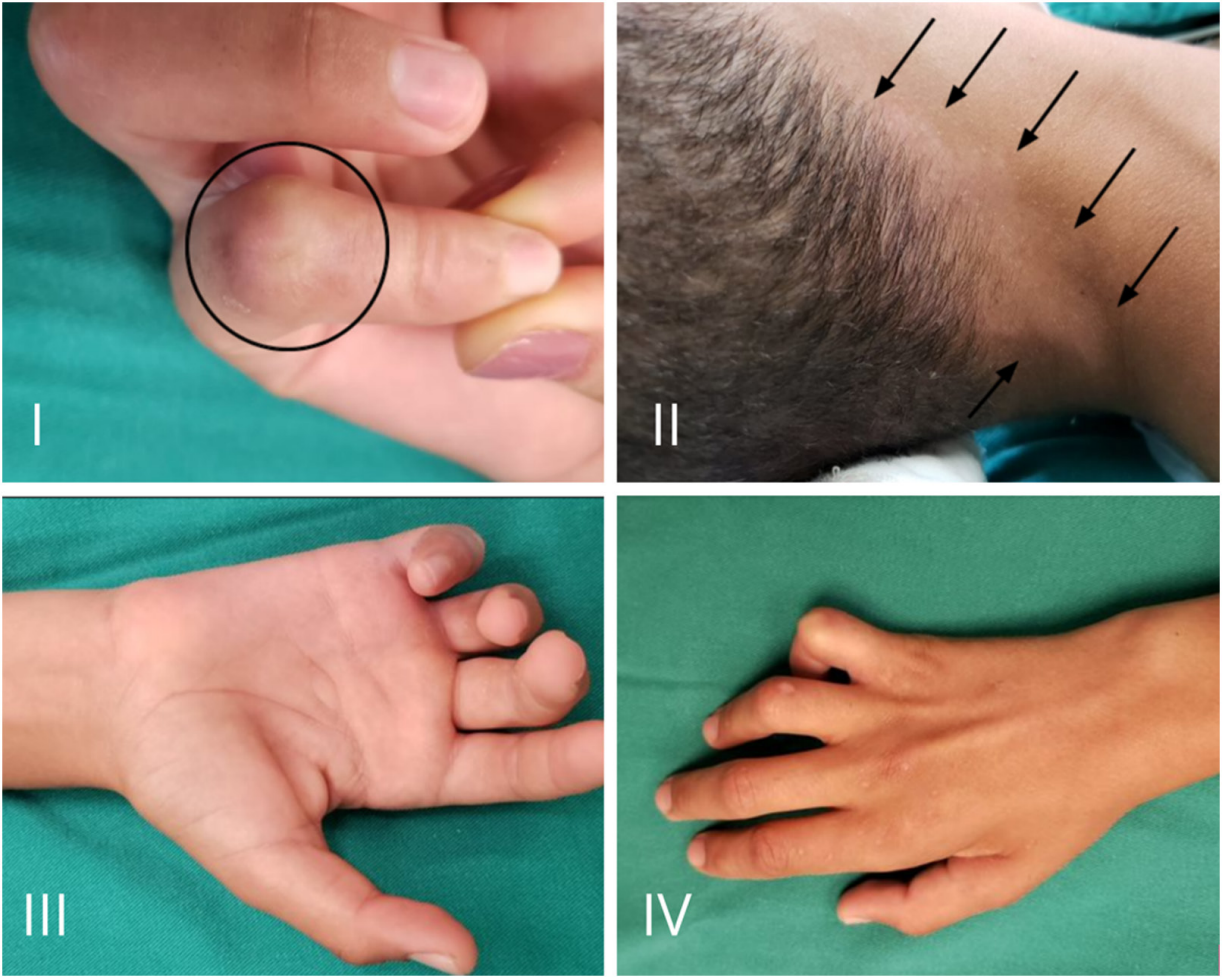
Scar on fifth finger secondary to candle burn. The minor did not notice the burn, nor even realize what had happened. (I) Hypochromic stain in the nape region on the right. (II) Palmar aspect of the right hand, showing claw deformity. (III) Dorsal aspect of the right hand, showing a claw deformity (IV).

We present a 13‐year‐old patient with a report of burn a year ago (Figure 1.I), who did not notice or feel it at the time. The injury was identified by the father, who referred him to the basic health service, where he was diagnosed with leprosy, occasion in which drug treatment was started according to the WHO protocol. He developed over 5 months with paresthesia on the medial aspect of the right forearm associated with progressive motor deficit and pain involving the elbow, forearm, and right wrist, in addition to the appearance of an ulnar claw, being then referred to our tertiary service specialized in neurosurgery.

On admission, he presented a hypochromic spot on the right nape and scalp region (Figure 1.II), which did not have thermal or tactile sensitivity. The test of the filaments with nylon showed loss of sensation, but still being able to feel deep pressure and pain in some regions, in the fourth and fifth fingers of the right hand (Orange filament). Motor deficit in the intrinsic muscles of the right hand (Figure 1.III and IV) and significant paresis of the deep flexor muscle of the fingers for the fourth and fifth fingers, with Grade 2 muscle strength, hypothenar atrophy, and positive Tinel's sign in the right elbow region were identified. Motor function of the median and radial nerves was preserved. Palpation showed a very thickened and painful right ulnar nerve in the elbow region.

**FIGURE 2:**
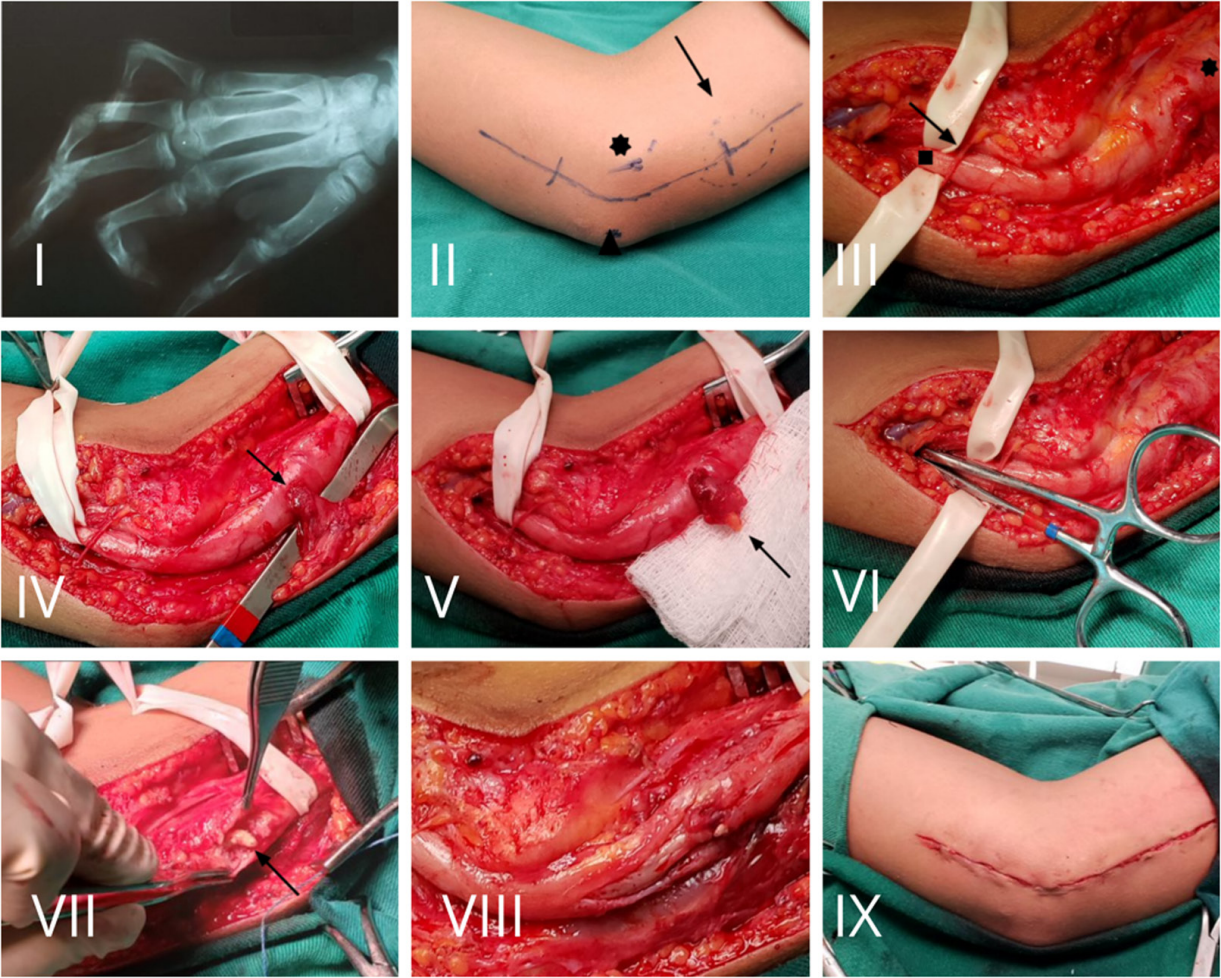
X‐ray of the right hand showing claw deformity. (I) Aspect of the surgical access marking. The medial epicondyle (Asterix) and the olecranon (triangle) are evident, as well as abnormal thickening of the ulnar nerve (arrow). (II) Aspect after external neurolysis and release of adhesions, and opening of Osborne's fascia. Note the ulnar nerve with significant thickening (star), distal stump with anatomical aspect (square), and a branch of the medial cutaneous nerve of the forearm crossing the ulnar nerve (arrow). (III) Presence of a plastron leaked by the ulnar nerve and adhered to the adjacent cell tissue (arrow). (IV) Aspect after sectioning the plastron from the adjacent tissue (arrow). (V) Significant distal decompression of the ulnar nerve. Evidenced by nonresistance passage of Kelly. (VI) After opening the epineurium, the presence of a large amount of caseous material inside the ulnar nerve was evidenced (arrow). (VII) Final aspect of the ulnar nerve, with an incised epineurium, after resection of all caseous material, showing free nervous fascicles and no intraneural mass effect. (VIII) Closing by planes. (IX) In the postoperative assessment period, the patient had improved the sensibility motor functions, without progress of the deformity.

An elbow US was performed, which showed an ulnar nerve with an increased caliber in the cubital tunnel region, suggesting granulomatous neuropathy. An x‐ray (Figure 2.I) was taken of the right hand, which showed rigid flexion of the fingers and signs of bone remodeling.

The patient was immediately referred for a surgical procedure for decompression of the right ulnar nerve.

## 3 SURGERY

Patient was placed in dorsal decubitus, under sedation and local anesthesia, with the right upper limb abducted and slightly flexed. A curvilinear incision (in Smile) was made in the anteromedial region of the elbow, between the olecranon and the medial epicondyle (Figure 2.II). The ulnar nerve was identified, quite thickened, with adhesions with all surrounding tissue.

It was released at the level of the tunnel between the olecranon and the medial epicondyle through the section of Osborne's fascia. External neurolysis of the ulnar nerve was performed until the distal and proximal segment was identified, which had a well‐thickened caliber. During the distal dissection of the ulnar nerve, the medial cutaneous nerve of the forearm, which traveled transversely to the ulnar nerve, was identified and preserved (Figure 2.III).

After external neurolysis, a thickened ulnar nerve with a whitish‐yellowish color was identified, in addition to the presence of fibrotic and fluid thickening, leaving the injured nerve and adhered to the adjacent tissue. This material was sectioned and resected, showing that it had a granulomatous and caseous content that flowed through the ulnar nerve (Figure 2.IV and V). There was evidence of sufficient free space for ulnar nerve decompression, both distally and medially (Figure 2.VI).

The ulnar nerve epineurium was then opened, showing a large amount of intraneural caseous material (Figure 2.VII), which was completely removed, relieving intraneural pressure and dissecting the space between the ulnar nerve fascicles (Figure 2.VIII).

The wound was exhaustively washed with 0.9% saline solution and then was closed in two planes, using 3‐0 nylon for skin (Figure 2.IX). After the procedure, the child was sent to the postanesthetic care room, being discharged to the ward after 2 h. The next day, he was discharged from the hospital.

## 4 COMMENT

Leprosy is a pathology that typically affects adults, with a latency time of 3–5 years; however, it can affect children, especially in places with low socioeconomic status. Some authors argue that this should be treated more like a neurological disease than a dermatological one, due to the flowery range of neurological manifestations.^7^

The rarity of neural form in children points to a situation of public calamity when identified. Early diagnosis and treatment must be promptly instituted to prevent deformities and disabilities for future adults.^8^, ^9^

Surgical decompression is an auxiliary method of treatment and prevention of irreversible neurological deformities and sequelae, and should be correctly and promptly indicated. In the case of neuropathy in children, conservative drug treatment can be tried for Stage I neuritis, but these children must be closely monitored.^5^, ^6^, ^10^

In this paper we present a rare case of Stage III right ulnar neuropathy in a 13‐year‐old child who underwent surgical decompression. Surgical approach of the caseous granuloma is the most appropriate treatment option for these patients, because of the advanced stage of the lesion. Although, recent studies call attention for the similarity with the use of conservative management.^11^

During the procedure, great thickening of the ulnar nerve was evidenced, and when epineurolysis was performed, the presence of abundant caseous granuloma was identified.

We did not find other such well‐documented cases of this surgical procedure in the literature. Pondé et al. described a case of a 6‐year‐old child who underwent surgery, but performed only the decompression of the ulnar in the bone tunnels. Lugão et al. described another case of an 11‐year‐old child who was decompressed and the surgery showed an abscess with a large amount of purulent secretion.

The findings in the literature, as well as those described in this study, emphasize the need for systematic and longitudinal care for patients during and after treatment, the period after discharge should not be neglected.

## AUTHOR CONTRIBUTIONS

**Fernando Henrique Morais de Souza:** Conceptualization; data curation; formal analysis; resources; software; supervision. **Luiz Euripedes Almondes Santana Lemos:** Funding acquisition; investigation; methodology; supervision; validation. **Bruno Rafael Sousa Rosado:** Data curation; formal analysis; funding acquisition; investigation; software; supervision; validation. **Raiza Rafaela Borges De Oliveira:** Conceptualization; data curation; formal analysis; investigation; methodology; resources; software; writing – original draft; writing – review and editing. **Mayle Gomes Ferreira de Araújo:** Formal analysis; methodology; project administration; visualization; writing – original draft; writing – review and editing. **Rhuann Pontes dos Santos Silva:** Formal analysis; investigation; resources; software. **Nivaldo Sena Almeida:** Data curation; formal analysis; methodology; software; supervision; writing – original draft. **Hildo Rocha Cirne Azevedo‐Filho:** Investigation; supervision.

## FUNDING INFORMATION

Not applicable.

## CONFLICT OF INTEREST STATEMENT

The authors have no conflict of interest to declare.

## CONSENT

Written informed consent was obtained from the patient to publish this report in accordance with the journal's patient consent policy.

## Data Availability

Not applicable.
